# Identification and Pathogenicity of *Fusarium* Species from Herbaceous Plants on Grassland in Qiaojia County, China

**DOI:** 10.3390/microorganisms13010113

**Published:** 2025-01-08

**Authors:** Yanzhu Gao, Zhixiao Zhang, Mei Ji, Sangzi Ze, Haodong Wang, Bin Yang, Lianrong Hu, Ning Zhao

**Affiliations:** 1 College of Biological Science and Food Engineering, Southwest Forestry University, Kunming 650224, China; gaoyanzhu98@163.com (Y.G.); 13038630242@163.com (H.W.); 2 Yunnan Academy of Forestry, Kunming 650224, China; zhangzhixiao@yafg.ac.cn (Z.Z.); jimei@yafg.ac.cn (M.J.); 3 Yunnan Forestry and Grassland Pest Control and Quarantine Bureau, Kunming 650224, China; zesangzi@163.com; 4 School of Biological and Chemical Science, Pu’er University, Pu’er 665000, China; yangbin48053@163.com; 5 Key Laboratory of Forest Disaster Warning and Control of Yunnan Province, Southwest Forestry University, Kunming 650224, China

**Keywords:** *Fusarium* complex species, *F. oxysporum*, gene locus, plant pathogens, molecular phylogeny

## Abstract

The *Fusarium* species is an important plant pathogen that can cause plant diseases in grassland, leading to the degradation of grassland quality. However, the morphology of *Fusarium* is greatly affected by environmental factors, which makes it difficult to identify its species. In addition, the pathogenic ability of different *Fusarium* species in plants has not been fully studied. In this study, *Fusarium* isolates were obtained from grassland herbaceous plants via tissue separation. Through morphological means and based on ITS, *RPB2*, and *TEF-1* gene sequences, we compared and constructed polygenic phylogenetic trees to classify and identify the *Fusarium* species. In addition, the pathogenicity of different *Fusarium* species was also analyzed. The results showed that a total of 24 *Fusarium* strains were successfully isolated from grassland, from which ten species were identified: *F. flagelliforme*, *F. longifundum*, *F. clavum*, *F. scirpi*, *F. ipomoeae, F. oxysporum*, etc. and were included in four complexes: *Fusarium incarnatum-equiseti* species complex (FIESC), *Fusarium oxysporum* species complex (FOSC), *Fusarium tricinctum* species complex (FTSC), and *Fusarium sambucinum* species complex (FSAMSC). Pathogenicity tests demonstrated that except for *F. ipomoeae* QJ5211, *F. sambucinum* QJ203, and *F. acuminatum* QJ1662, other *Fusarium* species had different degrees of pathogenic ability. This is the first study that discusses the effect of *Fusarium* on grassland disease control in this area. This study further provides clear pathogen information for the prevention and control of grassland diseases.

## 1. Introduction

Grasslands are an important part of the ecosystem and play an important role in protecting biodiversity [1,2,3]. However, grasslands are susceptible to fungal pathogens, among which *Fusarium* is a significant plant pathogen that can cause the slow rotting of leaves and roots in grasslands [4]. *Fusarium* often causes diseases on a number of Poaceae plants including rice, maize, wheat, barley, wild oat (*Avena fatua* L.), perennial ryegrass (*Lolium perenne* L.), etc., which can cause significant losses. Previous studies have found that *F. solani*, *F. oxysporum*, and *F. nivale* can lead to *Fusarium* wilt disease of perennial ryegrass [5,6,7]. In another study, Zhu found that *F. avenaceum*, *F. proliferatum*, and *F. chlamydosporum* can cause diseases in wild oat [8]. These *Fusarium* fungi are capable of destroying plant growth in different ways. On the one hand, *Fusarium* is capable of destroying the vascular system, which is crucial for the transport of water and minerals, thereby affecting the photosynthesis of the plant. In addition, *Fusarium* also produces mycotoxins that can destroy the cell cycle distribution, leading to mitochondrial dysfunction and inducing apoptosis, which ultimately leads to plant death [5,6]. These grasslands have good nutrition and can be used as feed for animals, especially wild oat and perennial ryegrass; therefore, it is particularly important to control herbal plant diseases in grasslands.

*Fusarium* is not merely a regional hazardous plant pathogen, but also poses significant harm to the international community and the economy, especially to some important economic crops. However, in the *Fusarium* genus, *F. oxysporum* has a strong pathogenic ability; it is a filamentous fungus of a facultative parasitic that can infect both plants and survive in the soil, colonizes the vascular system, and destroys the water-conducting xylem vessels, leading to plant wilt and death [9,10,11,12]. Furthermore, in the process of infection, *F. oxysporum* can induce a variety of virulence factors to destroy the host cells [13]. In addition, *F. oxysporum* can release a variety of toxins and cause the host plant to be pathogenic, thus leading to plant death [7,14] (e.g., banana blight caused by *F. oxysporum* f. sp. cubense (Foc) [15,16,17]; when the banana is infected by *Fusarium*, the plant xylem is damaged, causing the banana plant to die). Moreover, many studies have reported that *F. oxysporum* is also able to cause potato blight [18,19], pea blight [20], and chilli wilt. It is thus clear that *F. oxysporum* has a significant impact on many plants and crops, affecting the yield and quality. Moreover, *F. oxysporum* also affects human health, which was previously isolated from clinical practice by Debourgogne et al., who found that it could infect people’s skin, nails, eyes, and lungs, and that severe infection could even cause human death [21,22,23,24]. However, *F. graminearum*, *F. culmorum*, *F. pseudograminearum*, *F. avenaceum*, *F. equiseti*, and *F. poe* play an important role in cereal-like cash crops. Among them, *F. graminae* is the dominant pathogen that causes wheat crops to be infected with head blight. It can infect multiple parts of the host plant to cause plant disease, and three major global crops are especially sensitive to it: wheat, corn, and rice [25,26,27]. It is evident that *Fusarium* diseases are a serious threat to the yield and quality of crops. Therefore, understanding the characteristics and taxonomic status of the *Fusarium* species is important for the control of plant disease and human health.

The identification of the *Fusarium* species is mainly based on morphological characteristics [28]; however, because the morphology of *Fusarium* is highly affected by environmental factors, the classification framework of *Fusarium* has undergone major changes. Moreover, many *Fusarium* species are very similar in morphological characteristics, which makes them difficult to distinguish. With the development of molecular biology, it has become common to perform phylogenetic analyses combining genetic fragments of fungi to determine the taxonomic status of *Fusarium*. Current studies have reported many *Fusarium* complex species such as the *F. fujikuroi* species complex (FFSC), *F. incarnatum-equiseti* species complex (FIESC), *F. tricinctum* species complex (FTSC), *F. oxysporum* species complex (FOSC), and *F. sambucinum* species complex (FSAMSC) [28]. The classification of *Fusarium* complex species is difficult to undertake when only using an internal transcriptional spacer (ITS), therefore, it is crucial to combine multiple gene fragments to classify *Fusarium*.

Clarifying the taxonomic status and pathogenic potential of pathogens is the key to the effective prevention and control of plant diseases. In this study, we collected the leaves of symptomatic plants, obtained *Fusarium* by the tissue isolation method, used morphology combined with molecular biology to determine the taxonomic status of *Fusarium* species, and explored the pathogenicity of *Fusarium*. The results of the study can provide theoretical basis for the prevention and control of grassland diseases.

## 2. Materials and Methods

### 2.1. Isolation and Culture Conditions

A total of 269 grassland disease samples were collected in Qiaojia County (26°32′ N~27°25′ N, 102°52′ E~103°26′ E), Zhaotong City, Yunnan Province. Samples of diseased leaves were returned to the laboratory to isolate the fungi according to the tissue isolation method in [29] as follows. Leaves with disease were cleaned with sterile water, the moisture was absorbed with sterile filter paper, and a junction between the diseased tissue and healthy tissue was cut into a size measuring 0.5 cm × 0.5 cm. These were then disinfected with 75% (*v*/*v*) ethanol for 30 s, rinsed with sterile distilled water 3 times, dried with sterilized filter paper, and placed on potato dextrose agar (PDA) plates at 28 °C in the dark for 3–5 days.

### 2.2. Morphological Characteristics of Fusarium

After incubation in the dark on PDA plates for 7 days, the growth rates of all isolates was determined, and characteristics such as colony morphology, color, and shape were recorded [30]. A drop of sterile water was dropped onto the slide, and the cultures were taken from the surface of the pure cultured colonies and placed onto temporary glass slides under an optical microscope (ZEISS Axiocam ERc 5s, ZEISS, Oberkochen, Germany). The morphological characteristics of the mycelia were recorded. The shape, color, and size of the chlamydospore, sporulation cells, macroconidia, microconidia, and the spore size, among others, were measured, which included at least 30 data measurements for each strain.

### 2.3. DNA Extraction and Sequencing

Isolates were cultured on PDA plates at 28 °C for seven days. Mycelia were collected with a sterile inoculation shovel using the TSINGKE Plant DNA Extraction Kit (TSINGKE, Beijing, China) (Universal Type), and genomic DNA was extracted as well as three loci including ITS, *TEF-1*, and *RPB2*. The primers ITS5(GGAAGTAAAAGTCGTAACAAGG)/ITS4 (TCCTCCGCTTATTGATATGC) [31], EF1-728F(CATCGAGAAGTTCGAGAAGG)/EF4-986R (TACTTGAAGGAACCCTTACC) [32], and RPB2-6F (TGGGGYATGGTNTGYCCYGC) [33]/RPB2-7.1R (CCCATRGCYTGYTTMCCCATDGC) [34] were used to amplify the partial rDNA-ITS, *TEF-1*, and *RPB2* genes, respectively. The PCR thermal cycle program for ITS, *TEF-1*, and *RPB2* amplification was as follows: initial denaturation at 95 °C for 5 min, followed by 40 cycles of denaturation at 95 °C for 30 s, annealing at 54 °C for 30 s, elongation at 72 °C for 1 min, and a final extension at 72 °C for 10 min. Purification and sequencing of the PCR amplicons were undertaken by Tsingke (Beijing, China).

### 2.4. Phylogenetic Analysis

The obtained gene fragments were subjected to BLAST alignment analysis in NCBI (https://blast.ncbi.nlm.nih.gov/Blast.cgi, 24 September 2024) to find the closest related species and to refer its strain number to that found in other relevant studies. *Nectria eustromatica* (CBS 121896) and *Nectria mariae* (CBS 125294) were used as the outgroups [35]. In building the txt text, spping was performed using AliView software (AliView 1.28) to output the fasta format [36], while splicing was carried out with the Mesquite software (Mesquite 2.73) to output the fas format [37]. Two methods were used in the phylogenetic analyses: maximum likelihood (ML) and maximum parsimony (MP). Bootstrap replications were 5000; the model was based on the maximum composite likelihood. The maximum likelihood (ML) phylogenetic tree was constructed with raxmlGUI-2.0.0-beta [38] while the maximum parsimony (MP) phylogenetic tree was constructed with PAUPv4.0b10 software [39]. The maximum value was set to 5000, and bootstrap nreps was set to 1000. ML and MP analyses were performed through a heuristic search with MEGA11 [40]. The results were then processed through the FigTree 1.4 video speech tool [41]. The basic information of *Fusarium* used for phylogenetic analysis is shown in Supplementary Table S1.

### 2.5. Pathogenicity Tests

Fresh leaves of nine species of healthy disease-free plants were collected from Qiaojia County, Zhaotong City, Yunnan Province, China, for pathogenicity analysis: *Bromus japonicus* Thunb., *Capillipedium parviflorum* R., *Setaria viridis* L., *Themeda triandra* Forssk., *Heteropogon contortus* L., *Avena fatua* L., *Neotrinia splendens* Trin., *Brachypodium sylvaticum* Huds., and *Bothriochloa pertusa* L.

Before the pathogenicity tests with *Fusarium*, the *Fusarium* isolates were inoculated on fresh PDA medium in an incubator at 28 °C in dark conditions for 5–7 days. The fungi cakes were extracted with a 5 mm sterile punch, and a conidial suspension was subsequently prepared (1 × 10^6^ conidia/mL). Healthy and intact leaves were removed along the leaf sheaths for inoculation in vitro. Wounded and unwounded leaves were treated as follows. (i) Wounded leaves: The epidermal tissue of healthy plant was gently punctured with a sterile needle; the leaf wounds were inoculated with fungi cake and a conidial suspension of 5 μL, respectively. The damaged leaves were inoculated with cake lacking mycelium and sterile water as the controls, respectively. (ii) Unwounded leaves: The leaves were inoculated with fungi cake and a conidial suspension of 5 μL, respectively. Undamaged healthy leaves were inoculated with cake lacking mycelium and sterile water as the controls, respectively. Then, sterile wet cotton masses were placed on the side of the plant tissue for moisturizing and subsequently incubated for 24 h in a climate chamber of 25~28 °C under the condition of alternating light and dark for 12 h. Three leaves were placed onto each sterile Petri dish, and each treatment was performed in triplicate. Disease severity was measured 5 d after inoculation [42,43,44].

## 3. Results

### 3.1. Isolation and Identification of Fusarium Isolates

In 2022, a total of 24 *Fusarium* strains were isolated in 269 disease specimens in Qiaojia County, Yunnan Province. According to their morphological characteristics, the 24 studied isolates were divided into 10 morphological groups. The conidia of all *Fusarium* isolates were on PDA medium (Supplementary Table S2). For *F. sambucinum*, the colony was white, round, radial, and later secreted a pink and purple material (Supplementary Figure S1A,B). The chlamydospore was either single or serial (Supplementary Figure S1C). Macroconidia were sickle-shaped, slender, and curved, most of which consisted of three compartments, 17.3–31.1 µm × 3.5–4.8 µm (Supplementary Figure S1F). For *F. meridionale*, the colony was white to pink. Macroconidia were sickle- or rod-shaped, extending from the middle to both ends, gradually becoming pointed, 12.5–48.4 µm × 2.3–4.7 µm; microconidia were oval or near-oval, 3.6–9.4 µm × 2.2–3.1 µm (Supplementary Figure S2). For *F. avenaceum*, the colony was pink. Macroconidia were sickle-shaped, uniformly bent from the middle to both ends, 17.5–62.2 µm × 2.5–3.3 µm, while microconidia were kidney-shaped, cylindrical-shaped, or ovoid, 5.8–20.2 µm × 2.2–3.1 µm (Supplementary Figure S3). For *F. ipomoeae*, the colony was white. Conidia were sickle-shaped, 28.9–48.1 μm × 3.6–4.8 µm (Supplementary Figure S4). For *F. oxysporum,* the colony was purple. Conidia were slightly falcate-to-almost straight, oviform, or oval, 3.8–31.4 µm × 1.9–4.1 µm (Supplementary Figure S5). For *F. acuminatum*, the colony was pink, and the central surface of the colony in the late growth period was yellow. Conidia were slightly falcate-to-almost, oval, or nearly elliptic, 8.2–35.4 µm × 2.6–4.1 µm (Supplementary Figure S6). For *F. longifundum*, the colony gas hyphae were more developed, initially white villous and gradually produced a yellow material. Macroconidia were transparent scyshaped, straight, or shoot curved, apical cell microcurved, papillary, basal cell microcurved, foot-shaped, 33.3–56.6 μm × 3.1–4.6 μm; microconidia were kidney, crescent, or sickle, 9.6–18.0 μm × 1.9–3.1 μm (Supplementary Figure S7). For *F. clavum*, the colony was white to yellow. The conidiophore was lateral or terminal, having a single vial stem or co-vial stem, with branching. Macroconidia were straight, sickle-shaped, 19.1–44.1 µm × 3.1–5.9 µm, while microconidia were oval or nearly cylindrical, 6.8–15.6 µm × 2.5–4.1 µm (Supplementary Figure S8). For *F. sambucinum*, the colony was white. Chlamydospores were spherical to ellipsoid. Conidiogenous cells had a single vial stem or compound vial stem with tree branches. Macroconidia were transparent scyshaped, straight, or curved, apical cells were microcurved, 15.9–37.5 µm × 2.8–3.7 µm, while the microconidia were oval, crescent, or cylindrical in shape, 5.9–16.5 µm × 2.5–3.1 µm (Supplementary Figure S9). For *F. scirpi*, the colony was creamy white, and the mycelium flocculent. The chlamydospore was single or serial, colorless to brown. The macroconidia falciform was slightly straight in the middle, and curved at both ends, 3.0–4.7 µm × 29.3–53.3 µm, while the microconidia were ovate to nearly oval, 2.9–3.3 µm × 5.6–20.3 µm (Supplementary Figure S10).

In this study, the morphological characteristics of *Fusarium* were basically consistent with those reported [6,8,28,45,46,47,48]. However, compared to other *Fusarium* species, research on *F. flagelliforme*, *F. longifundum*, and *F. scirpi* is limited. Therefore, the discovery of *Fusarium* is conducive to expanding the library of fungal resources and laying the foundation for *Fusarium* related research.

### 3.2. Multigene Phylogenetic Analysis

**Figure 1:**
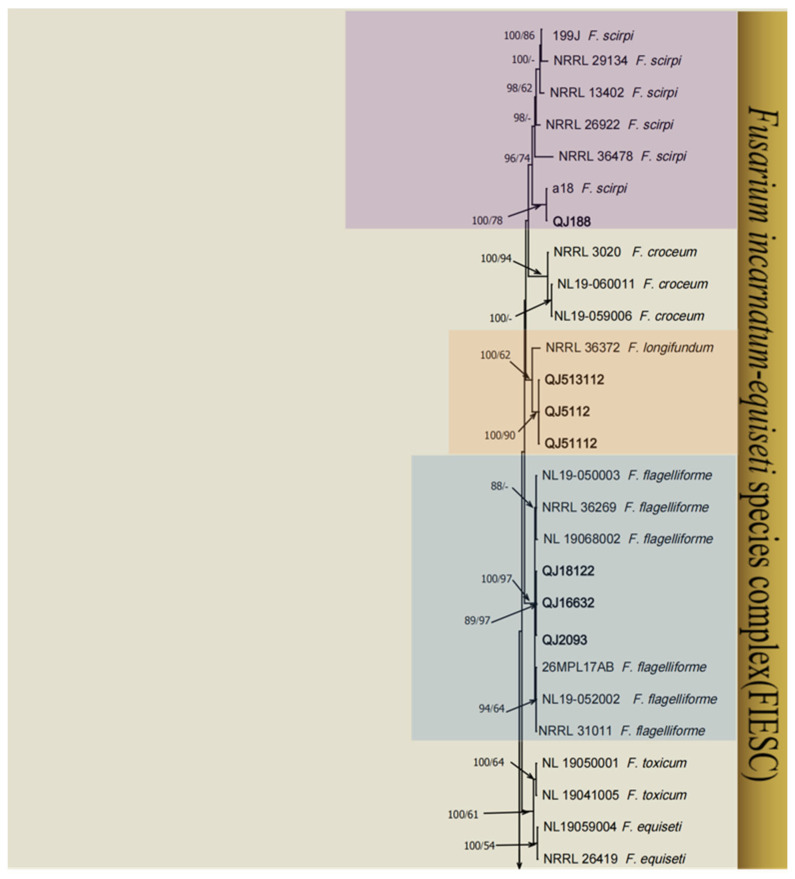

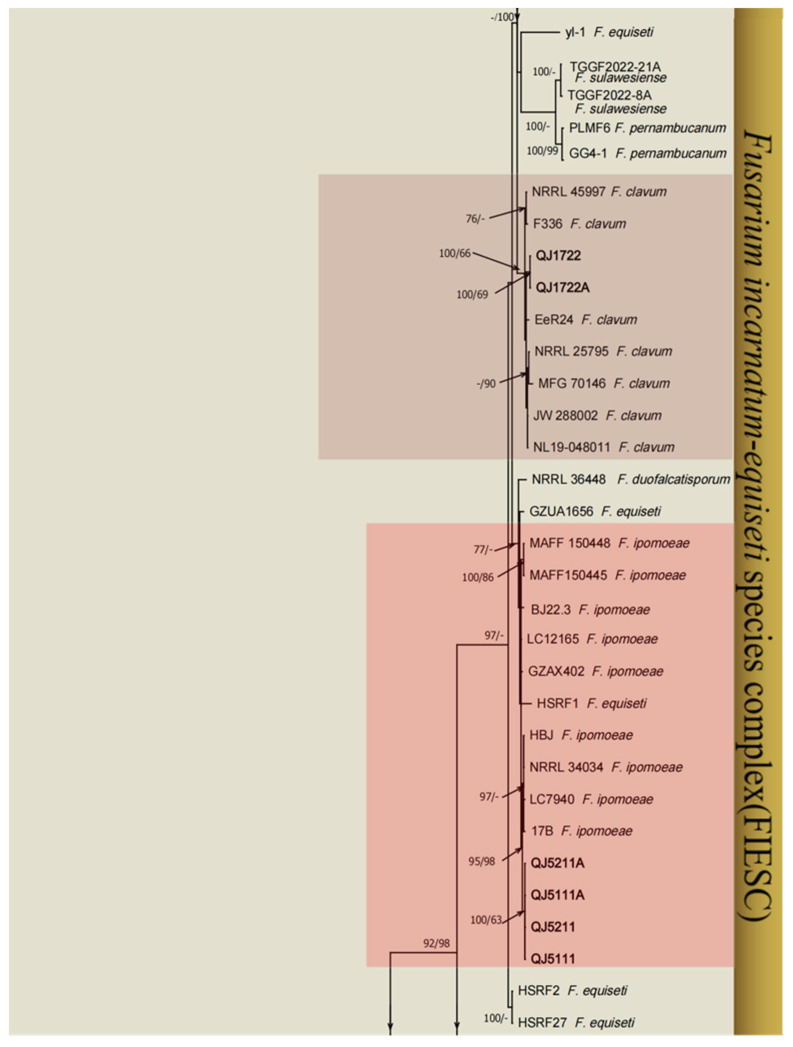
Maximum likelihood (ML) phylogenetic analysis based on the DNA sequence data from the *ITS*, *TEF-1*, and *RPB2* loci of *Fusarium* spp. From the *Fusarium incarnatum-equiseti* species complex (FIESC). Support values at the nodes represent the ML and MP bootstrap percentages, with values greater than or equal to 70% and 50% shown at the nodes. Numbers in the nodes represent the support in bootstrap analyses (1000 replications). Different background colors represent different *Fusarium* species. The arrows at the top and bottom of the figure indicate connections to the figure below, and the slanted arrows in the center indicate he values of ML and MP from left to right, respectively. *Fusarium* isolates used for the study are indicated in bold. The tree was rooted in two outgroup sequences of *N. eustromatica* and *N. mariae*.

**Figure 2:**
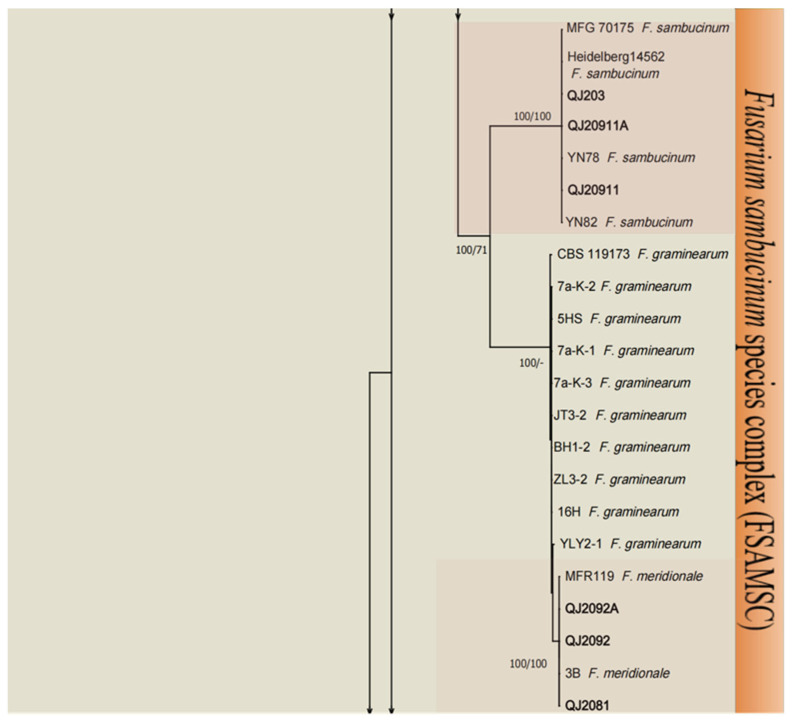
Maximum likelihood (ML) phylogenetic analysis based on the DNA sequence data from the *ITS*, *TEF-1*, and *RPB2* loci of *Fusarium* spp. From the *Fusarium sambucinum* species complex (FSAMSC). Support values at the nodes represent the ML and MP bootstrap percentages, with values greater than or equal to 70% and 50% shown at the nodes. Numbers in the nodes represent the support in bootstrap analyses (1000 replications). Different background colors represent different *Fusarium* species. The arrows at the top and bottom of the figure indicate connections to the figure below. *Fusarium* isolates used for the study are indicated in bold. The tree was rooted in two outgroup sequences of *N. eustromatica* and *N. mariae*.

**Figure 3:**
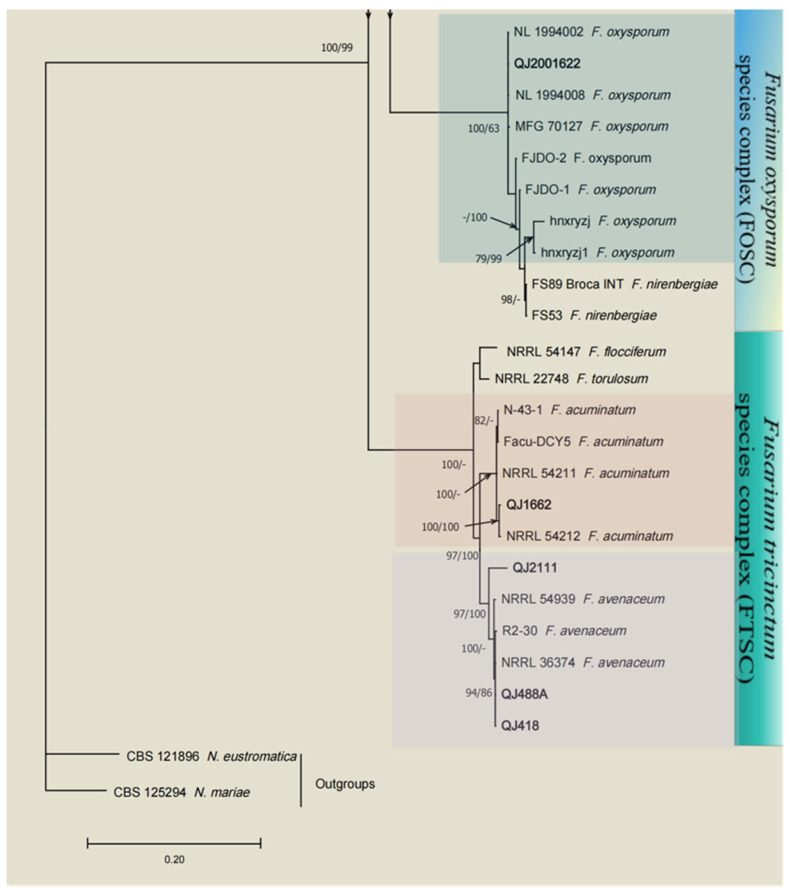
Maximum likelihood (ML) phylogenetic analysis based on the DNA sequence data from the *ITS*, *TEF-1*, and *RPB2* loci of *Fusarium* spp. From the *Fusarium oxysporum* species complex (FOSC) and *Fusarium tricinctum* species complex (FTSC). Support values at the nodes represent the ML and MP bootstrap percentages, with values greater than or equal to 70% and 50% shown at the nodes. Numbers in the nodes represent the support in bootstrap analyses (1000 replications). Different background colors represent different *Fusarium* species. The arrows at the top and bottom of the figure indicate connections to the figure below, and the slanted arrows in the center indicate he values of ML and MP from left to right, respectively. *Fusarium* isolates used for the study are indicated in bold. The tree was rooted in two outgroup sequences of *N. eustromatica* and *N. mariae*.

A total of 24 *Fusarium* strains were successfully isolated from the leaves of diseased herbs, and to investigate the taxonomic status of these isolates, we conducted a multilocus analysis of the ITS, *TEF-1*, and *RPB2* sequences to infer their relatedness among the *Fusarium* strains. The total *Fusarium* species phylogeny included 106 sequences including two outgroup sequences of *N. eustromatica* (CBS 121896) and *N. mariae* (CBS 125294). In the multilocus phylogenetic analysis, the tree showed that 24 strains of *Fusarium* were assigned to four *Fusarium* complex species: the *F. incarnatum-equiseti* species complex (FIESC), *F. sambucinum* species complex (FSAMSC), *F. oxysporum* species complex (FOSC), and *F. tricinctum* species complex (FTSC). These four *Fusarium* complex species were analyzed in combination with their morphological characteristics, and these isolates were identified in five species in FIESC (*F. flagelliforme*, *F. longifundum*, *F. clavum*, *F. scirpi* and *F. ipomoeae*) (Figure 1), two species in FSAMSC (*F. sambucinum* and *F. meridionale*) (Figure 2), one species in FOSC (*F. oxysporum*) (Figure 3), and two species in FTSC (*F. acuminatum* and *F. avenaceum*) (Figure 3). The most abundant of the above four species was FIESC.

### 3.3. Pathogenicity of Fusarium Isolates

In order to explore the pathogenicity of *Fusarium* isolates to grassland plants, the pathogenicity of isolates was explored according to the results of the phylogenetic analysis. The pathogenicity of *Fusarium* isolates is shown in Supplementary Table S3.

**Figure 4:**
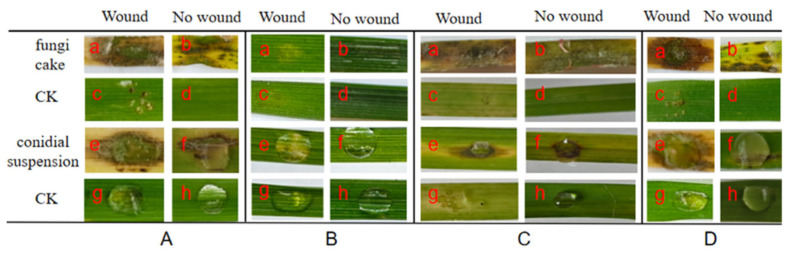
Symptoms of the leaves at 5 days after inoculation of the *Fusarium tricinctum* species complex (FTSC). (**A**) Pathogenicity of *F. avenaceum* QJ418 to *Avena fatua* L. (**B**) Pathogenicity of *F. acuminatum* QJ1662 to *Neotrinia splendens* Trin. (**C**) Pathogenicity of *F. avenaceum* QJ2111 to *Brachypodium sylvaticum* Huds. (**D**) Pathogenicity of *F. avenaceum* QJ488A to *Avena fatua* L. (**a**) Wound, inoculation fungi cake. (**b**) No wound, inoculation fungi cake. (**c**) Wound, without fungi cake. (**d**) No wound, without fungi cake. (**e**) Wound, inoculation conidial suspension. (**f**) No wound, inoculation conidial suspension. (**g**) Wound, inoculation sterile water. (**h**) No wound, inoculation sterile water.

The results of the pathogenicity experiments showed that most of the strains showed different degrees of symptoms after 5 days of inoculation, except for *F. ipomoeae* QJ5211, *F. sambucinum* QJ203, and *F. acuminatum* QJ1662, which were not pathogenic. In FTSC, except for *F. acuminatum,* which did not have a pathogenic ability to *Neotrinia splendens* Trin. (Figure 4B), *F. avenaceum* had strong pathogenicity against two different hosts, *Avena fatua* L. and *Brachypodium sylvaticum* Huds., after 5 days of the inoculation of the in vitro leaves; the experimental group had symptoms in both the wounded and unwounded leaves, while the control group had no symptoms. Moreover, the leaves wilted in the inoculated group 5 days later, where the pathogenicity of these two *Fusarium* species was 100% (Figure 4A,C,D).

**Figure 5:**
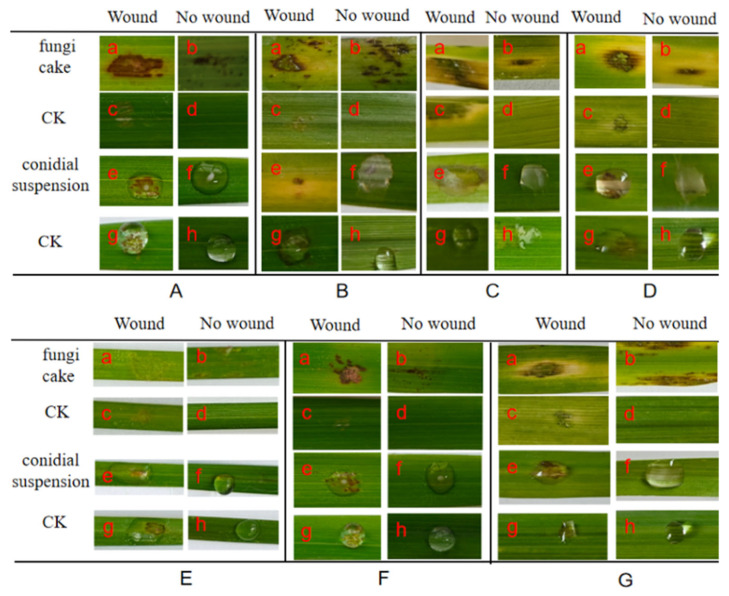
Symptoms of the leaves at 5 days after inoculation of the *Fusarium sambucinum* species complex (FSAMSC) and *Fusarium oxysporum* species complex (FOSC). (**A**–**F**) The pathogenicity of FSAMSC. (**A**) Pathogenicity of *F. meridionale* QJ2092 to *Capillipedium parviflorum* R. (**B**) Pathogenicity of *F. meridionale* QJ2081 to *Setaria viridis* L. (**C**) Pathogenicity of *F. sambucinum* QJ20911 to *Avena fatua* L. (**D**) Pathogenicity of *F. sambucinum* QJ20911A to *Avena fatua* L. (**E**) Pathogenicity of *F. sambucinum* QJ203 to *Bothriochloa pertusa* L. (**F**) Pathogenicity of *F. meridionale* QJ2092A to *Capillipedium parviflorum* R. (**G**) Pathogenicity of *F. oxysporum* QJ2001622 from FOSC to *Avena fatua* L. (**a**) Wound, inoculation fungi cake. (**b**) No wound, inoculation fungi cake. (**c**) Wound, without fungi cake. (**d**) No wound, without fungi cake. (**e**) Wound, inoculation conidial suspension. (**f**) No wound, inoculation conidial suspension. (**g**) Wound, inoculation sterile water. (**h**) No wound, inoculation sterile water.

In FSAMSC, except for *F. sambucinum* QJ203, which was not pathogenic to *Bothriochloa pertusa* L., the other five isolates were pathogenic, and the inoculated control leaves showed no symptoms. No symptoms were observed in the inoculated control leaves (Figure 5). The pathogenicity of *F. sambucinum* to *Avena fatua* L. was 10–60% (Figure 5C,D; Supplementary Table S3), while the pathogenicity of *F. meridionale* to *Setaria viridis* L. was 33.3–88.9% (Figure 5B; Supplementary Table S3). The pathogenicity of *F. meridionale* to *Capillipedium parviflorum* R. was 50–100% (Figure 5A,F; Supplementary Table S3). In FOSC, after inoculation with the *F. oxysporum* fungi cake, the leaves showed symptoms including a yellowish brown color and leaf discoloration whereas the control group (CK) remained asymptomatic (Figure 5G).

**Figure 6:**
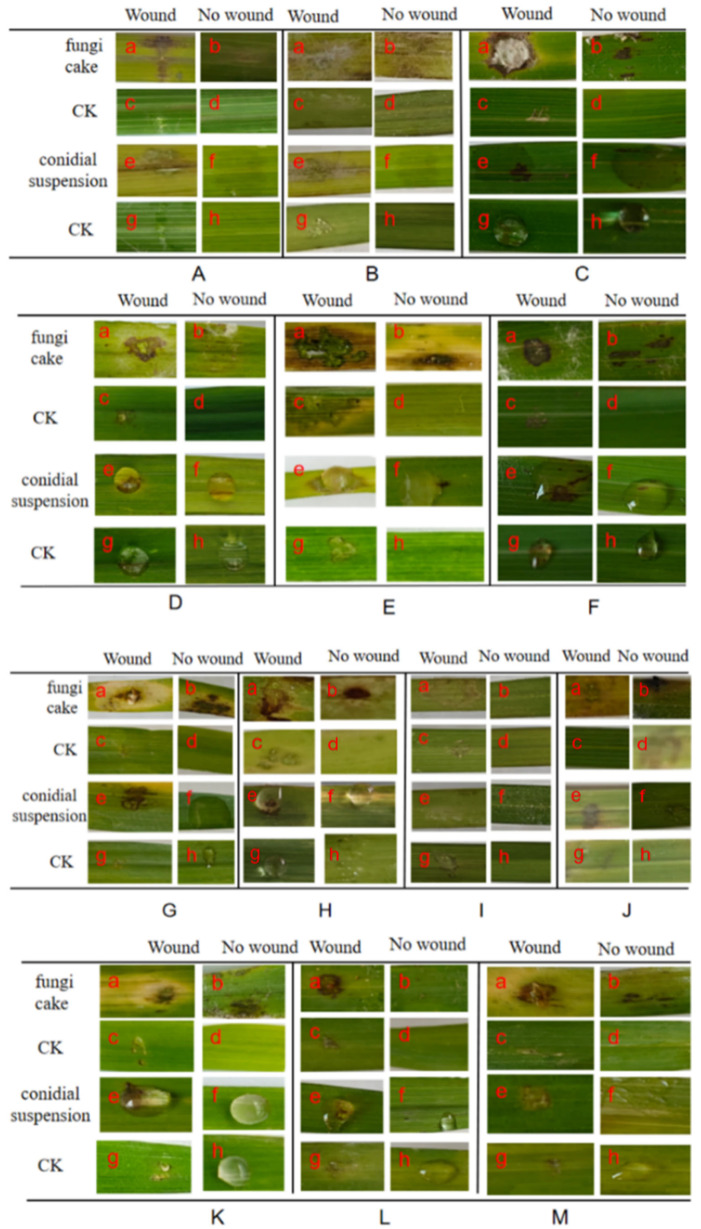
Symptoms of the leaves at 5 days after inoculation of the *Fusarium incarnatum-equiseti* species complex (FIESC). (**A**) Pathogenicity of *F. clavum* QJ1722 to *Themeda triandra* Forssk. (**B**) Pathogenicity of *F. clavum* QJ1722A to *Themeda triandra* Forssk. (**C**) Pathogenicity of *F. scirpi* QJ188 to *Capillipedium parviflorum* R. (**D**) Pathogenicity of *F. flagelliforme* QJ18122 to *Bromus japonicus* Thunb. (**E**) Pathogenicity of *F. flagelliforme* QJ16632 to *Avena fatua* L. (**F**) Pathogenicity of *F. flagelliforme* QJ2093 to *Capillipedium parviflorum* R. (**G**) Pathogenicity of *F. ipomoeae* QJ5111 to *Setaria viridis* L. (**H**) Pathogenicity of *F. ipomoeae* QJ5111A to *Setaria viridis* L. (**I**) Pathogenicity of *F. ipomoeae* QJ5211 to *Heteropogon contortus* L. (**J**) Pathogenicity of *F. ipomoeae* QJ5211A to *Heteropogon contortus* L. (**K**) Pathogenicity of *F. longifundum* QJ513112 to *Avena fatua* L. (**L**) Pathogenicity of *F. longifundum* QJ5112 to *Setaria viridis* L. (**M**) Pathogenicity of *F. longifundum* QJ51112 to *Setaria viridis* L. (**a**) Wound, inoculation fungi cake. (**b**) No wound, inoculation fungi cake. (**c**) Wound, without fungi cake. (**d**) No wound, without fungi cake. (**e**) Wound, inoculation conidial suspension. (**f**) No wound, inoculation conidial suspension. (**g**) Wound, inoculation sterile water. (**h**) No wound, inoculation sterile water.

In FIESC, except for *F. ipomoeae* QJ5211, which is not pathogenic to *Heteropogon contortus* L. (Figure 6I), the other 12 isolates had a pathogenic ability. No symptoms were observed in the inoculated control leaves (Figure 6). *F. clavum* was inoculated with *Themeda triandra* Forssk. Five days later, except for the uninjured conidial suspension treatment group, the leaves of the other treatment groups were light yellow-brown, and none of the control groups had any disease (Figure 6A,B). The pathogenicity of *F. clavum* to *Themeda triandra* Forssk. was 10–50% (Supplementary Table S3). After inoculation with *F. scirpi*, the leaves showed dark brown symptoms (Figure 6C). The pathogenicity of *F. scirpi* to *Capillipedium parviflorum* R. was 33.3–100% (Supplementary Table S3). *F. flagelliforme* had a strong pathogenic ability and could cause symptoms in the leaves of *Bromus japonicus* Thunb., *Capillipedium parviflorum* R., and *Avena fatua* L., with the strongest pathogenicity reaching 100% (Figure 6D–F; Supplementary Table S3). After treatment with *F. ipomoeae*, the leaves of *Setaria viridis* L. showed symptoms, with brown in the middle and yellow edges (Figure 6G,H), and its pathogenicity was 50–100% (Supplementary Table S3). The pathogenicity of *F. longifundum* to *Avena fatua* L. and *Setaria viridis* L. was 77.8–100% and 10–90%, respectively (Figure 6K–M; Supplementary Table S3).

## 4. Discussion

In this study, a total of 24 *Fusarium* strains were isolated from grasslands including four *Fusarium* complex species, FIESC (*F. flagelliforme*, *F. longifundum*, *F. clavum*, *F. scirpi* and *F. ipomoeae*), FOSC, FTSC (*F. acuminatum*, and *F. avenaceum*), and FSAMSC (*F. sambucinum* and *F. meridionale*) via a multiphylogenetic analysis of the ITS, *TEF1*, and *RPB2* loci. The *Fusarium* species have some morphological features of their own; because of the influence of multiple factors such as the environment and host, species identification needs to be performed via macroscopic and microscopic analyses [49,50]. Since most *Fusarium* species rarely form sexual periods on a common medium, their morphology is usually classified by macroconidia, microconidia, chlamydospores, etc., in the asexual period. Molecular identification has high specificity and sensitivity, and it accelerates the detection of plant pathogens. Currently, many genes have been used in the identification of *Fusarium* species using the PCR amplification of DNA, and the frequently used gene loci include tubulin (*TUB*), ITS, *TEF-1*, calmodulin (*CAM*), *RPB2,* etc. [51,52]. Previously, the molecular identification of *Fusarium* mainly relied on the phylogenetic analysis of a single locus (ITS or *TEF-1*) [53]. However, with the combined use of several marker genes and sequencing platforms, the knowledge of *Fusarium* taxa has increased [54].

*Fusarium* will not only cause the wilt and decay of plant roots, stems, and leaves, resulting in crop reduction, grassland area reduction, and quality decline, but also produces some secondary metabolites and mycotoxins. Many species have been reported in *Fusarium* toxins such as trichothecenes (TSs), searalenonethe, butenolide, moniliformin, deoxynivalenol, fumonisins, deoxynivalenol (DON) [55], nivalenol, the estrogenic mycotoxin zearalenone (ZEA) [56], fumonisin (FB), alternata toxin (AAL), etc. [57,58]. TS is a sesquiterpene compound composed of multiple fusion rings that is produced by *F. graminearum*, *F. culmorum*, *F. sporotrichioides*, *F. poae*, and *F. solani*, which have similar structures, can endanger animal health, and can cause serious diseases and even death. Trichothecene is one of the most harmful toxins of mycotoxins, which is toxic to animals and plants, and has a significant impact on the production of grain cash crops [10]. It can be phytotoxic to wheat, leading to the production of plant peroxides, which affects its defense response and causes the yellowing of wheat, reducing its yield and quality [59]. In addition, the compound has dramatic effects on humans, causing symptoms of vomiting, nausea, anorexia, abdominal pain, growth inhibition, diarrhea, bleeding, and immunotoxicity [60,61]. It severely threatens human health and affects the quality and safety of agricultural products and food [62,63]. FB and AAL with strong toxicity can kill plant nuclei and can cause liver and kidney failure and cancer when consumed by animals or humans [64]. Moreover, *Fusarium* is able to produce the toxin fusaric acid, which can reprogram the host metabolic pathways and induce senescence to increase its severity [20]. Therefore, it is crucial to clarify the classification and secondary metabolite species of different *Fusarium* for plant disease control. The production and virulence of secondary metabolites in *Fusarium* isolates should be further investigated in the follow-up of this study.

In this paper, eight pigment-producing *Fusarium* strains were isolated including all strains of FTSC and FSAMSC, two complex species. Fungal pigments are an important class of compounds with diverse colors, variety, and functions. The study found that some pigments play an important role in antibacterial, anticancer, antiviral, and other aspects, for example, the pigments produced by *F. graminearum* are able to inhibit yeast growth [65]. *F. oxysporum* produces pink-to-purple anthraquinone pigments [66], which are a form of widely used natural pigments that are pollution-free and play an important role in food and medicine [67]. Leslie and Summerell identified a slow-growing *F. acuminatum* with carmine pigmentation [68]; Fanelli et al. also found that *F. acuminatum* could produce red or brown pigments in agar [6]. Although many studies have been reported on *Fusarium* pigments, we only observed whether pigments were produced in this study, and the research on the pigment-producing ability of *Fusarium* and the specific species of pigments needs to be further explored.

*Fusarium* species can be classified into pathogenic and non-pathogenic *Fusarium* species, and not all *Fusarium* species are pathogenic. Saito et al. successfully isolated two non-pathogenic *Fusarium* species, *F. commune* and *F. proliferatum*, which were able to reduce the infection of rice seeds [69]. *F. oxysporum,* as an important pathogenic fungus, poses a serious threat to many plants, but this fungus also has non-pathogenic strains. The authors of [70] reported a non-pathogenic *F. oxysporum*, *F. oxysporum*Fo47, isolated from French soil and is a natural inhibitor of blight in tomato and melon; in addition, many researchers have continuously reported the biological control potential of non-pathogenic *F. oxysporum* [71,72,73,74]. In this study, it was concluded that FIESC was the most isolated *Fusarium* in grassland diseases in Qiaojia County including five species. Han et al. also reported FIESC, among which about half of the *Fusarium* came from cereals [28]. Therefore, it is very important to explore the pathogenicity of FIESC for the prevention and control of gramineous grasses and cereal diseases. The results of the pathogenicity assessment showed that in addition to *F. acuminatum* and *F. sambucinum*, other *Fusarium* were pathogenic, and *F. avenaceum* had a strong pathogenic ability to different hosts. Therefore, *F. avenaceum* can be considered as an important pathogen when designing and implementing disease management protocols.

It can be seen that pathogenic *Fusarium* poses a major threat to gramineous herbage. Therefore, it is of great significance to pay attention to *Fusarium* disease in future research for the development of grassland construction and animal husbandry, and strengthening the research on the pathogenicity of *Fusarium* is more conducive to the healthy development of cash crops and grassland resources. This study not only provides a basis for the prevention and control of grassland diseases, but also provides a reference for the resource utilization of the *Fusarium* species. However, further exploration is needed to explore the pathogenicity of *Fusarium* on different grassland vegetation.

## 5. Conclusions

Multilocus phylogenetic analysis was performed using ITS, *TEF-1*, and *RPB2* sequences. These *Fusarium* isolates were identified as follows: five species in FIESC (*F. flagelliforme*, *F. longifundum*, *F. clavum*, *F. scirpi*, and *F. ipomoeae*), two species in FSAMSC (*F. sambucinum* and *F. meridionale*), one species in FOSC (*F. oxysporum*), and two species in FTSC (*F. acuminatum* and *F. avenaceum*). This is the first time that the *Fusarium* disease has been reported in Qiaojia County. Finally, pathogenicity tests confirmed the pathogenicity of the *Fusarium* isolates.

## Data Availability

The data presented in this study have been deposited in the GenBank repository. The accession numbers can be found in the article. The data are included in the article and Supplementary Materials.

## Supplementary Materials

The following supporting information can be downloaded at: https://www.mdpi.com/article/10.3390/microorganisms13010113/s1, Table S1: Details of the *Fusarium* strains included in the phylogenetic analyses; Table S2: Conidia characteristics on potato dextrose agar (PDA) of the *Fusarium* isolates; Table S3: Pathogenicity of the *Fusarium* isolates; Supplementary Figure S1: Morphological characteristics of *F. sambucinum*; Supplementary Figure S2: Morphological characteristics of F. meridionale; Supplementary Figure S3: Morphological characteristics of *F. avenaceum*; Supplementary Figure S4: Morphological characteristics of *F. ipomoeae*; Supplementary Figure S5: Morphological characteristics of *F. oxysporum*; Supplementary Figure S6: Morphological characteristics of *F. acuminatum*; Supplementary Figure S7: Morphological characteristics of *F. longifundum*; Supplementary Figure S8: Morphological characteristics of *F. clavum*; Supplementary Figure S9: Morphological characteristics of *F. flagelliforme*; Supplementary Figure S10: Morphological characteristics of *F. scirpi*.
