# Supplementary material for: Identification and Pathogenicity of Fusarium Species from Herbaceous Plants on Grassland in Qiaojia County, China
Source: Microorganisms. 2025 Jan 8;13(1):113. doi: 10.3390/microorganisms13010113 (PMC11767762; doi:10.3390/microorganisms13010113)
Supplement: Supplementary file 1 [file microorganisms-13-00113-s001.zip › Revised Supplementary Material Figure1-10.pdf]

microorganisms

MDPI

Article

Identification and Pathogenicity of Fusarium Species from Herbaceous Plants on Grassland in Qiaojia County, China

Yanzhu Gao[1], Zhixiao Zhang[2], Mei Ji[2], Sangzi Ze[3], Haodong Wang[1], Bin Yang[4], Lianrong Hu[2,*], Ning Zhao[1,5,*]

[1]College of Biological Science and Food Engineering, Southwest Forestry University, Kunming 650224, China gaoyanzhu98@163.com (Y.G.); 13038630242@163.com (H.W.)
[2]Yunnan Academy of Forestry, Kunming 650224, China; jimei@yafg.ac.cn (M.J.); zhangzhixiao@yafg.ac.cn (Z.Z.)
[3]Yunnan Forestry and Grassland Pest Control and Quarantine Bureau, Kunming 650224, China; zesangzi@163.com (S.Z.)
[4]School of Biological and Chemical Science, Pu'er University, Pu'er 665000, China; yangbin48053@163.com (B.Y.)
[5]Key Laboratory of Forest Disaster Warning and Control of Yunnan Province, Southwest Forestry University, Kunming 650224, China
* Correspondence: hulianrong@yafg.ac.cn (L.H.); lijiangzhn@swfu.edu.cn (N.Z.)

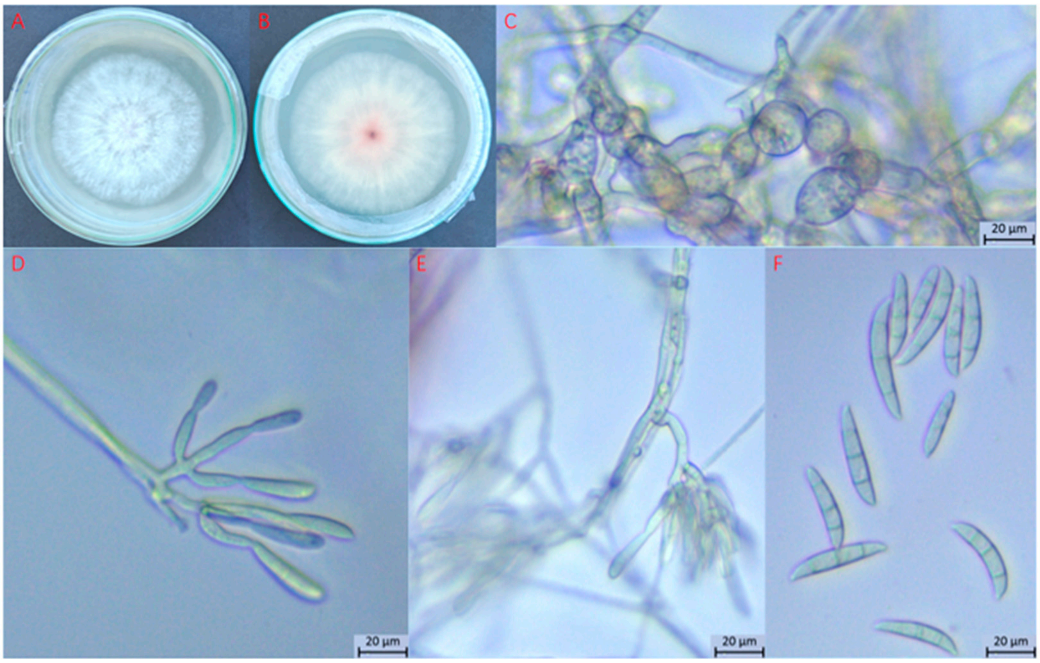

Supplementary Figure 1. Morphological characteristics of *F. sambucinum*. **A-B:** Colonies after 4 days on PDA; **A:** Above; **B:** Reverse; **C:** Chlamydospore; **D-E:** Generator cell; **F:** Conidia.

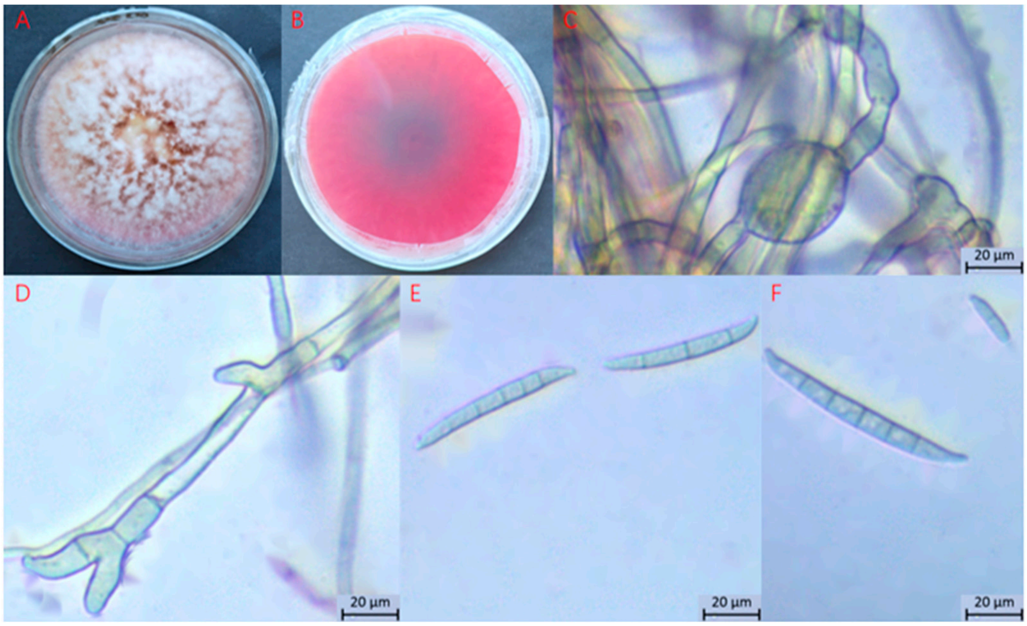

Supplementary Figure 2. Morphological characteristics of *F. meridionale*. **A-B:** Colonies after 7 days on PDA; **A:** Above; **B:** Reverse; **C:** Chlamydospore; **D:** Pycnidia; **E-F:** Conidia.

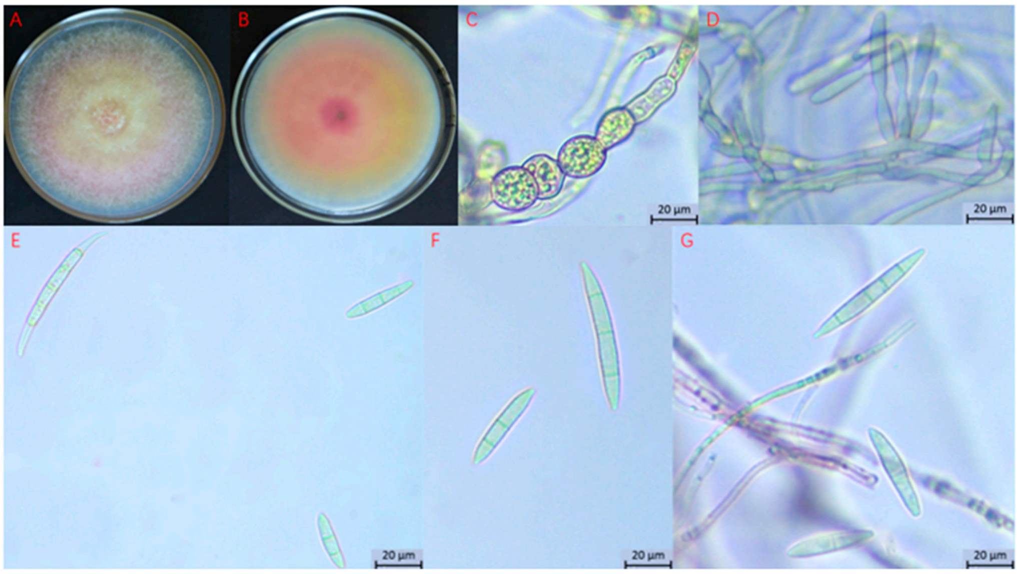

Supplementary Figure 3. Morphological characteristics of *F. avenaceum*. **A-B:** Colonies after 7 days on PDA; **A:** Above; **B:** Reverse; **C:** Chlamydospore; **D:** Pycnidia; **E-G:** Conidia.

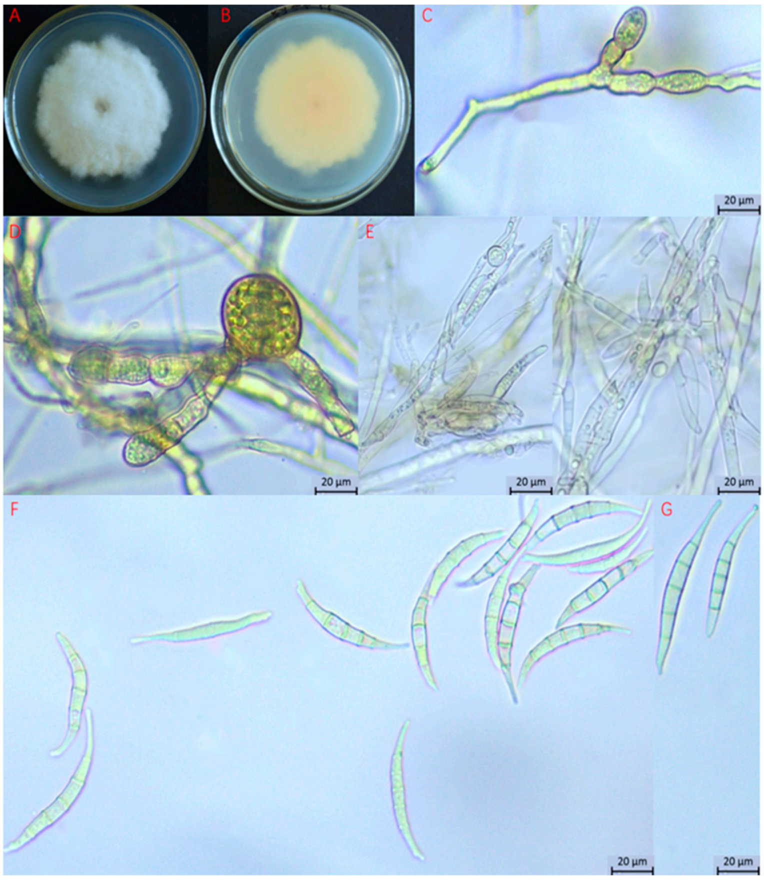

Supplementary Figure 4. Morphological characteristics of *F. ipomoeae*. **A-B:** Colonies after 6 days on PDA; **A:** Above; **B:** Reverse; **C-D:** Chlamydospore; **E:** Pycnidia; **F-G:** Conidia.

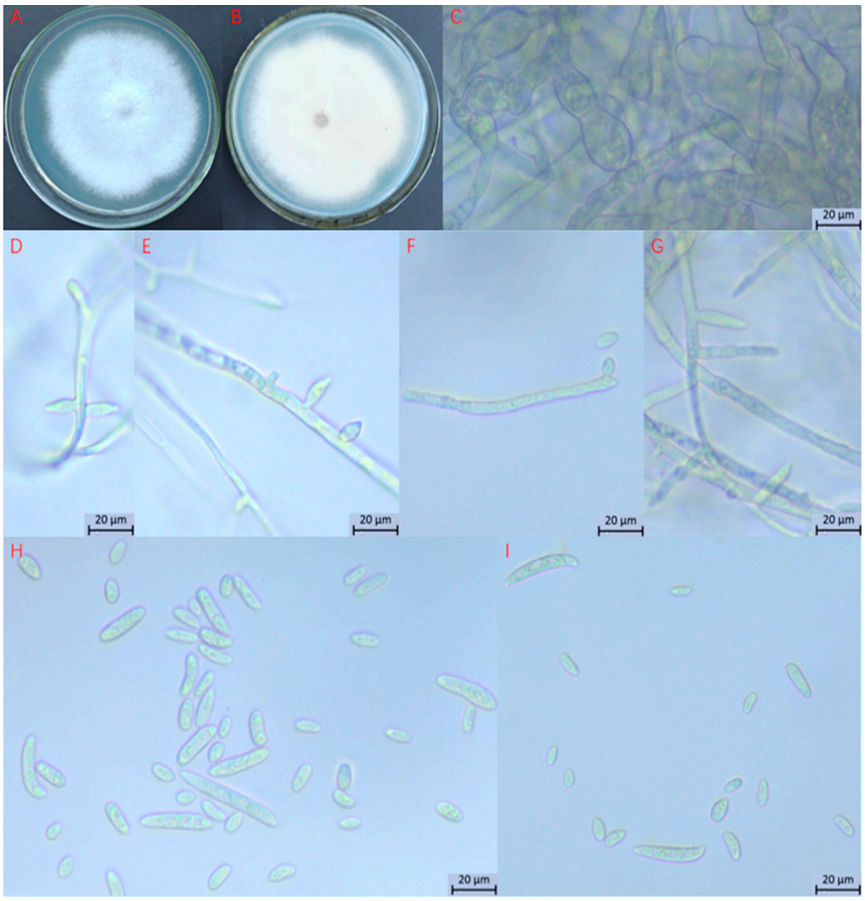

Supplementary Figure 5. Morphological characteristics of *F. oxysporum*. **A-B:** Colonies after 7 days on PDA; **A:** Above; **B:** Reverse; **C:** Chlamydospore; **D-G:** Generator cell; **H-I:** Conidia.

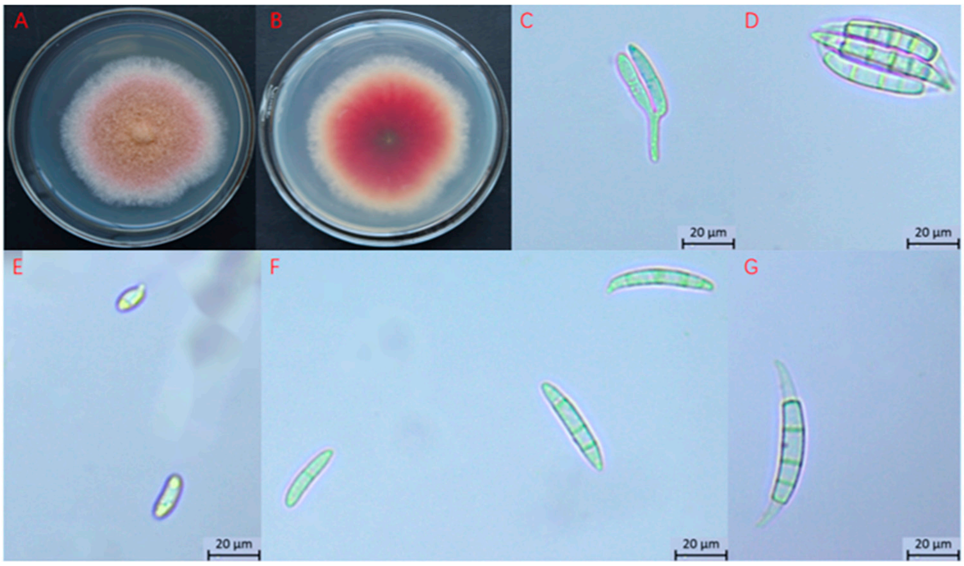

Supplementary Figure 6. Morphological characteristics of *F. acuminatum*. **A-B:** Colonies after 5 days on PDA; **A:** Above; **B:** Reverse; **C-D:** Chlamydospore; **E-G:** Conidia.

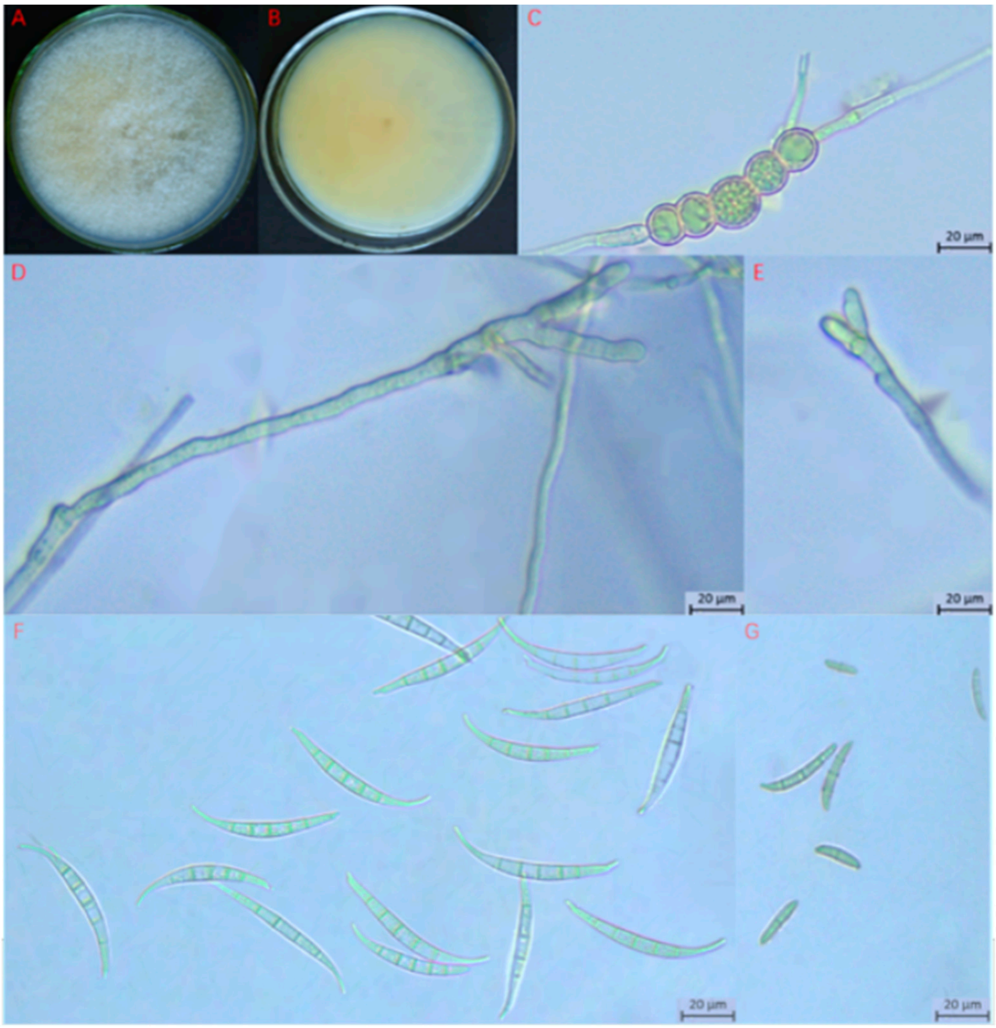

Supplementary Figure 7. Morphological characteristics of *F. longifundum*. **A-B:** Colonies after 7 days on PDA; **A:** Above; **B:** Reverse; **C:** Chlamydospore; **D-E:** Generator cell; **F-G:** Conidia.

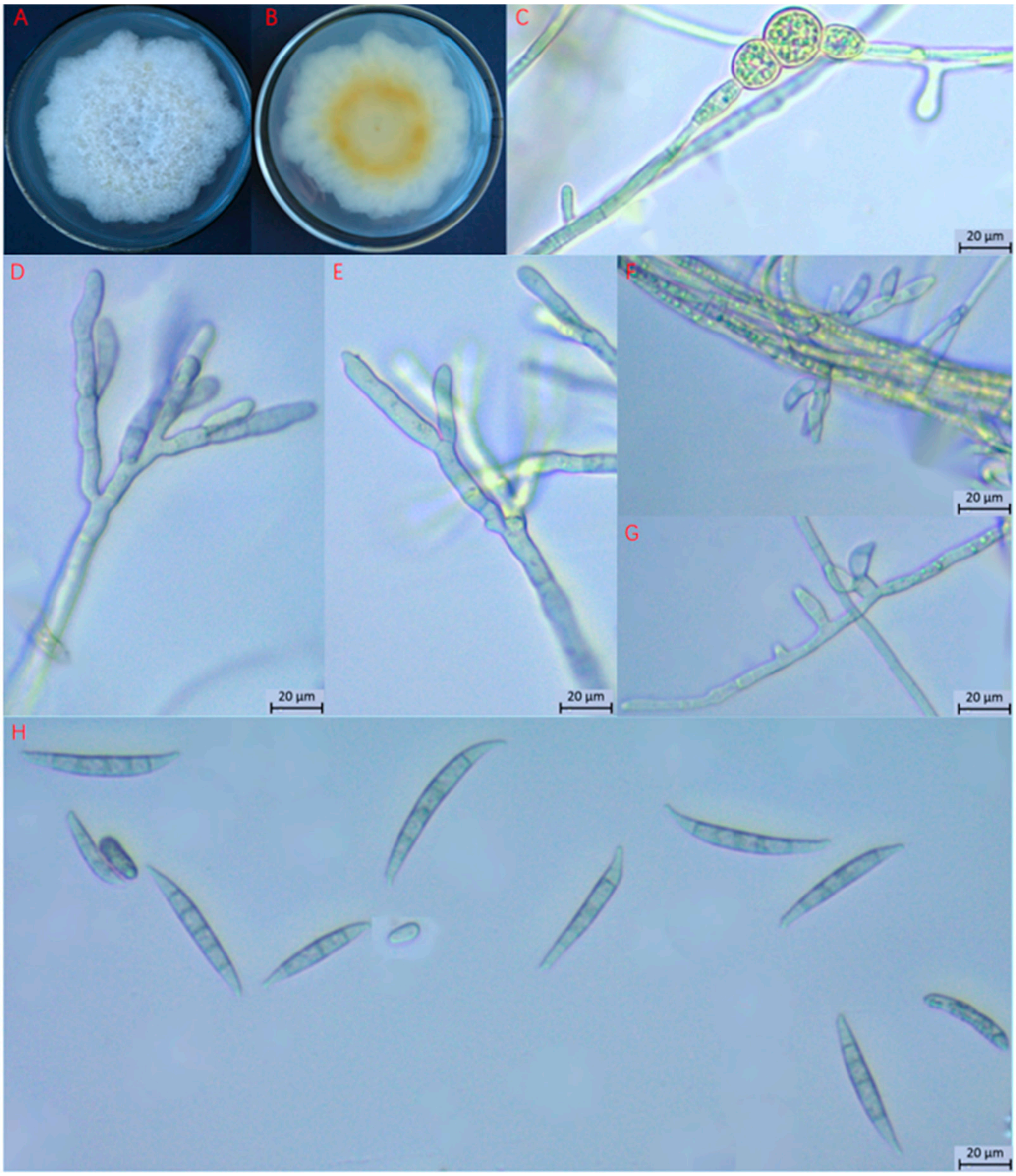

Supplementary Figure 8. Morphological characteristics of *F. clavum*. **A-B:** Colonies after 6 days on PDA; **C:** Chlamydospore; **D-G:** Conidiophore; **H:** Macroconidia and microconidia.

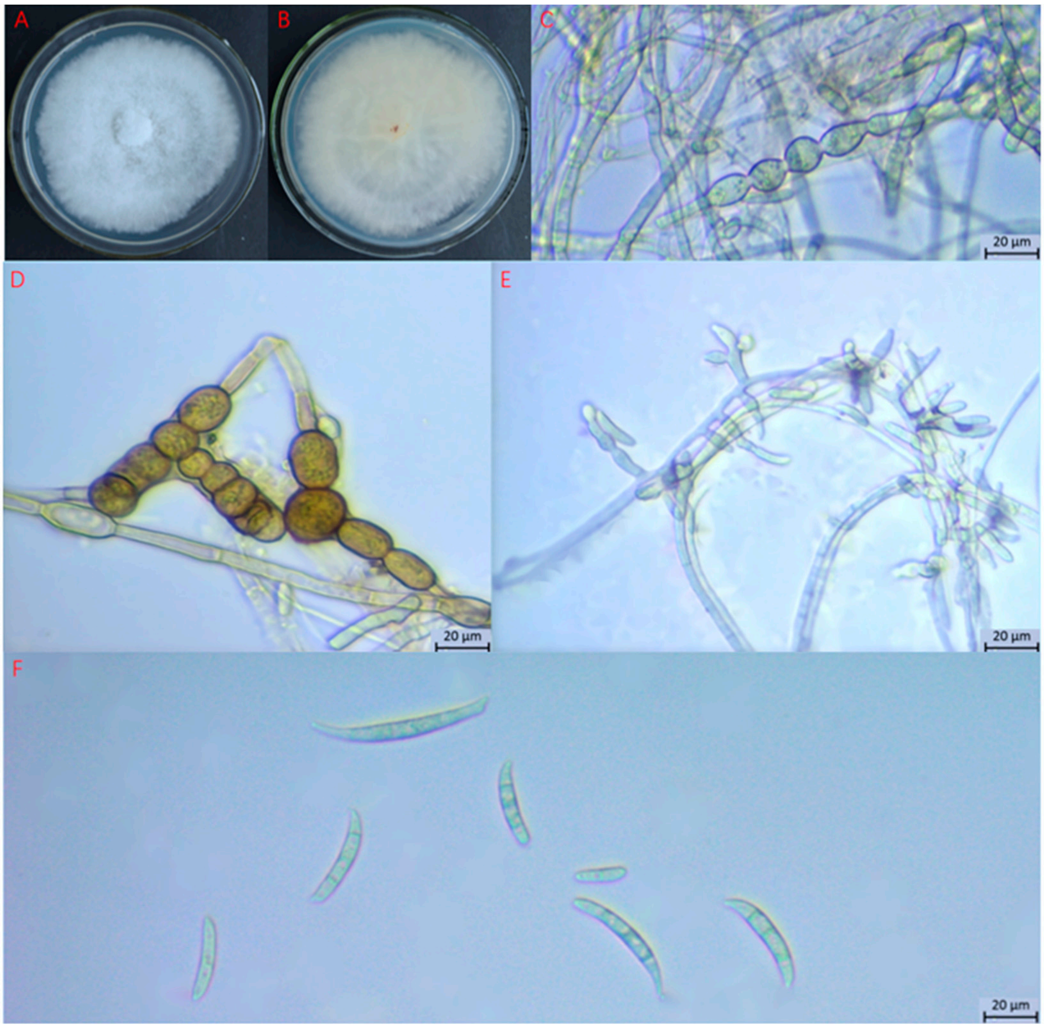

Supplementary Figure 9. Morphological characteristics of *F. flagelliforme*. **A-B:** Colonies after 7 days on PDA; **A:** Above; **B:** Reverse; **C-D:** Chlamydospore; **E:** Conidiogenous cells; **F:** Conidia.

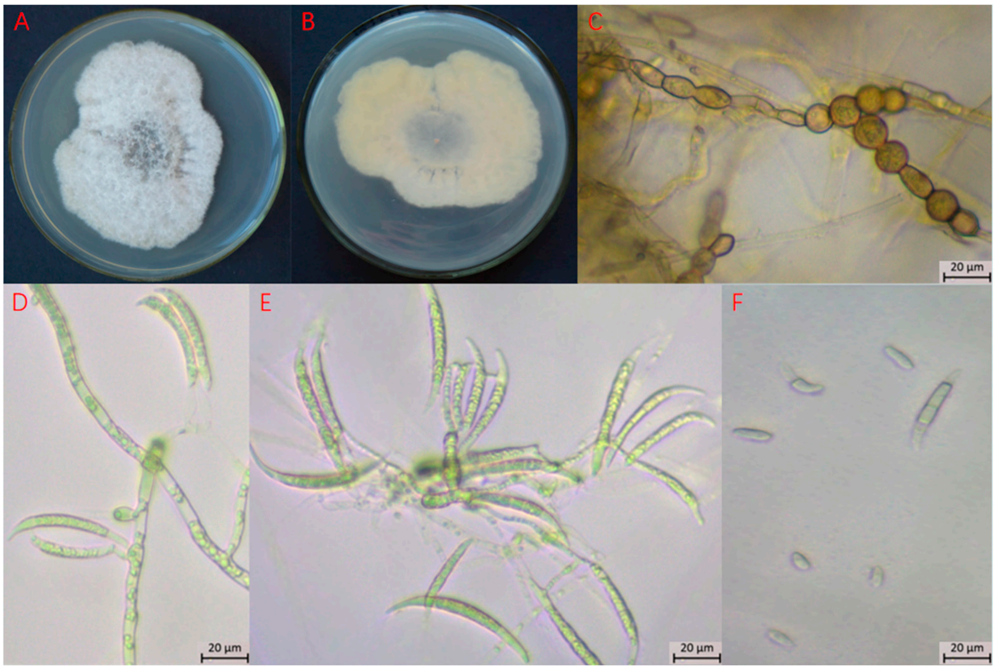

Supplementary Figure 10. Morphological characteristics of *F. scirpi*. **A-B:** Colonies after 7 days on PDA; **A:** Above; **B:** Reverse; **C:**

Chlamydospore; **E:** macroconidia; **F:** microconidia.
