# Supplementary material for: Identification and Pathogenicity of Fusarium Species from Herbaceous Plants on Grassland in Qiaojia County, China
Source: Microorganisms. 2025 Jan 8;13(1):113. doi: 10.3390/microorganisms13010113 (PMC11767762; doi:10.3390/microorganisms13010113)
Supplement: Supplementary file 1 [file microorganisms-13-00113-s001.zip › Revised Supplementary Material Table S1-S3.pdf]

microorganisms

MDPI

Article

Identification and Pathogenicity of *Fusarium* Species from Herbaceous Plants on Grassland in Qiaojia County, China

Yanzhu Gao[1], Zhixiao Zhang[2], Mei Ji[2], Sangzi Ze[3], Haodong Wang[1], Bin Yang[4], Lianrong Hu[2,*], Ning Zhao[1,5,*]

[1]College of Biological Science and Food Engineering, Southwest Forestry University, Kunming 650224, China gaoyanzhu98@163.com (Y.G.); 13038630242@163.com (H.W.)
[2]Yunnan Academy of Forestry, Kunming 650224, China; jimei@yafg.ac.cn (M.J.); zhangzhixiao@yafg.ac.cn (Z.Z.)
[3]Yunnan Forestry and Grassland Pest Control and Quarantine Bureau, Kunming 650224, China; zesangzi@163.com (S.Z.)
[4]School of Biological and Chemical Science, Pu'er University, Pu'er 665000, China; yangbin48053@163.com (B.Y.)
[5]Key Laboratory of Forest Disaster Warning and Control of Yunnan Province, Southwest Forestry University, Kunming 650224, China
* Correspondence: hulianrong@yafg.ac.cn (L.H.); lijiangzhn@swfu.edu.cn (N.Z.)

Table S1. Details of *Fusarium* strains included in the phylogenetic analyses.

Species name	Accession number	GenBank accession no.		
		ITS	TEF1	RPB2
F. acuminatum	N-43-1	MT560377		
F. acuminatum	Facu-DCY5	—	ON331997	ON331995
F. acuminatum	NRRL 54211	HM068319	HM068309	HM068329
F. acuminatum	**QJ1662**	PQ289632	PQ421085	PQ227790
F. acuminatum	NRRL 54212	HM068320	—	HM068330
F. avenaceum	**QJ2111**	PQ295808	PQ337245	PQ337257
F. avenaceum	NRRL 54939	PP336534	OL772892	OL773196
F. avenaceum	**QJ418**	PQ295809	PQ337246	PQ337258
F. avenaceum	**QJ488A**	PQ295810	PQ337247	PQ337259
F. avenaceum	R2-30	LC724043	LC724044	LC724046
F. avenaceum	NRRL 36374	OL832183	OL772755	OL773059
F. clavum	EeR24	ON908966	OP293703	OP293705
F. clavum	**QJ1722**	PQ289631	PQ280811	PQ227791
F. clavum	**QJ1722A**	PQ295799	PQ337248	PQ337260
F. clavum	F336	OR123399	OQ511147	OR371943
F. clavum	NRRL 45997	GQ505761	GQ505672	GQ505850
F. clavum	MFG 70146	—	OR634794	OR727723
F. clavum	NRRL 25795	GQ505686	GQ505597	—
F. clavum	JW 288002	MZ890484	MZ921826	MZ921694
F. clavum	NL19-048011	MZ890486	MZ921828	MZ921696
F. croceum	NRRL 3020	GQ505675	GQ505586	GQ505764
F. croceum	NL19-060011	MZ890490	MZ921832	MZ921700

F. croceum	NL19-059006	MZ890489	MZ921831	MZ921699
F.duofalcatisporum	NRRL 36448	GQ505741	GQ505652	GQ505830
F. equiseti	GZUA.1656	MG839500	MG839497	MG839491
F. equiseti	HSRF1	OR473084	OR475297	PP118283
F. equiseti	yl-1	MN150474.1	MN128581	MK848689
F. equiseti	HSRF27	OR473103	OR528674	PP118271
F. equiseti	HSRF2	OR473085	OR475298	PP118284
F. equiseti	NRRL 26419	GQ505688	GQ505599	GQ505770
F. equiseti	NL19-059004	MZ890494	MZ921837	MZ921705
F. flagelliforme	26MPL17AB	—	ON292364	—
F. flagelliforme	NL19-050003	MZ890498	MZ921841	MZ921709
F. flagelliforme	NL19-052002	MZ890499	MZ921842	MZ921710
F. flagelliforme	NRRL 31011	GQ505695	GQ505606	GQ505784
F. flagelliforme	NL19-068002	MZ890500	MZ921843	MZ921711
F. flagelliforme	NRRL 36269	GQ505734	GQ505645	GQ505823
F. flagelliforme	**QJ2093**	PQ295807	PQ325565	PQ337251
F. flagelliforme	**QJ16632**	PQ295798	PQ325563	PQ337249
F. flagelliforme	**QJ18122**	PQ295800	PQ325564	PQ337250
F. flocciferum	NRRL 54147	—	—	MH582369
F. torulosum	NRRL 22748	—	—	MH582376
F. graminearum	CBS 119173	—	KT855178	KT855204
F. graminearum	YLY2-1	—	OP10520	OP785265
F. graminearum	5HS	—	MZ07899	MZ07895
F. graminearum	7a-K-3	OR598801	OR604580	PP576702
F. graminearum	7a-K-2	OR598800	OR604579	PP576701
F. graminearum	7a-K-1	OR598799	OR604578	PP576700
F. graminearum	ZL3-2	—	OP105205	OP785266
F. graminearum	JT3-2	—	OP105200	OP785261
F. graminearum	16H	MT185454	MT238998	MT250617
F. graminearum	BH1-2	—	OP105195	OP785256
F. ipomoeae	LC7940	MK280798	MK289642	K289796
F. ipomoeae	17B	MT185457	MT239001	MT250620
F. ipomoeae	HBJ	MZ409522	MZ41153	MZ411535
F. ipomoeae	NRRL 34034	GQ505725	GQ505636	GQ505814
F. ipomoeae	**QJ5211A**	PQ295816	PQ348088	PQ337264
F. ipomoeae	**QJ5211**	PQ295815	PQ348087	PQ337263
F. ipomoeae	**QJ5111**	PQ295811	PQ348085	PQ337261
F. ipomoeae	**QJ5111A**	PQ295813	PQ348086	PQ337262
F. ipomoeae	GZAX 402	OP454871	OP432881	OP432883
F. ipomoeae	LC12165	MK280832	MK28959	MK28975
F. ipomoeae	BJ22.3	MT946880	MT946880	MT946881

F. ipomoeae	MAFF 150448	LC685056	LC685060	LC685189
F. ipomoeae	MAFF 150445	LC685055	LC685059	LC685188
F. longifundum	**QJ5112**	PQ295814	PQ348090	PQ337253
F. longifundum	**QJ513112**	PQ295817	PQ348091	PQ337254
F. longifundum	**QJ51112**	PQ295812	PQ348089	PQ337252
F. longifundum	NRRL 36372	GQ505738	GQ505649	GQ505827
F. meridionale	MFR119	—	ON316722	N502983
F. meridionale	3B	OR538625	OR553042	OR561908
F. meridionale	**QJ2081**	PQ295802	PQ348092	PQ325566
F. meridionale	**QJ2092**	PQ295805	PQ348093	PQ325567
F. meridionale	**QJ2092A**	PQ295806	PQ348094	PQ325568
F. nirenbergiae	FS53	OR538687	OR553051	OR561970
F. nirenbergiae	FS89 Broca INT	OR538698	OR553057	OR561981
F. oxysporum	hnxryzj1	OP071248	OP087208	OP467559
F. oxysporum	hnxryzj	ON872218	ON897740	OP467557
F. oxysporum	FJDO-1	MK880499	MW546934	MZ031970
F. oxysporum	FJDO-2	MK880500	MW546935	MZ031971
F. oxysporum	MFG 70127	—	OR634785	OR727736
F. oxysporum	**QJ2001622**	PQ295818	PQ348095	PQ337256
F. oxysporum	NL19-94002	MZ890538	MZ921884	MZ921753
F. oxysporum	NL19-94008	MZ890539	MZ921885	MZ921754
F. pernambucanum	PLMF6	—	LC800437	LC810990
F. pernambucanum	GG4-1	—	PP102795	PP102807
F. sambucinum	MFG 70175	—	OR020730	OR727753
F. sambucinum	YN82	—	OR019814	OR019826
F. sambucinum	YN78	—	OR019813	OR019825
F. sambucinum	**QJ20911**	PQ295803	PQ348096	PQ337265
F. sambucinum	**QJ203**	PQ289633	PQ421086	PQ227792
F. sambucinum	**QJ20911A**	PQ295804	PQ348097	PQ337266
F. sambucinum	Heidelberg14562	MZ707296	MZ707294	MZ707295
F. scirpi	a18	—	PP420214	OR859740
F. scirpi	**QJ188**	PQ295801	PQ348098	PQ337255
F. scirpi	NRRL 26922	GQ505690	GQ505601	GQ505779
F. scirpi	NRRL 13402	GQ505681	GQ505592	GQ505770
F. scirpi	NRRL 29134	GQ505694	GQ505605	GQ505783
F. scirpi	199J	—	PP874558	PP874507
F. scirpi	NRRL 36478	GQ505743	GQ505654	GQ505832
F. sulawesiense	TGGF2022-8A	—	OR427939	OR509691
F. sulawesiense	TGGF2022-21A	—	OR427944	OR509696
F. toxicum	NL19-050001	MZ890504	MZ921847	MZ921715
F. toxicum	NL19-041005	MZ890502	MZ921845	MZ921713

Nectria eustromatica	CBS 121896	HM534896	HM534875	HM534886
Nectria mariae	CBS 125294	MH863507	—	KM232404

—: GenBank accession no. are not available in other studies.
Fusarium isolates used for phylogenetic analysis are indicated in bold.

Table S2. Conidia characteristics on potato dextrose agar (PDA) of *Fusarium* isolates.

Fusarium species	Isolate	conidia		
		Length(μm)	width(μm)	Septa
F. acuminatum	QJ1662	9.6-56.6	1.9-4.6	0-5
F. avenaceum	QJ211	5.8-62.2	2.2-3.3	0-6
F. avenaceum	QJ418	13.2-36.6	1.6–3.8	0-5
F. avenaceum	QJ488A	18.5-39.4	2.8-3.9	1-4
F. oxysporum	QJ2001622	3.8-31.4	1.9–4.1	0-4
F. sambucinum	QJ20911	18.1-23.8	3.4-4.6	0-4
F. sambucinum	QJ203	17.3-31.1	3.5-4.8	0-5
F. sambucinum	QJ20911A	15.3-29.8	2.8-4.6	0-5
F. meridionale	QJ2081	10.7-39.8	2.6-3.9	0-4
F. meridionale	QJ2092	9.4-48.4	2.3-4.7	0-5
F. meridionale	QJ2092A	7.8-39.9	2.2-4.3	0-5
F. flagelliforme	QJ16632	5.9-47.3	2.5-3.1	0-6
F. flagelliforme	QJ18122	6.1-40.9	1.9-3.5	0-5
F. flagelliforme	QJ2093	8.0-46.9	2.6-3.9	0-5
F. longifundum	QJ51112	9.6-56.6	1.9-4.6	1-5
F. longifundum	QJ5112	9.8-54.9	2.4-4.8	0-5
F. longifundum	QJ513112	7.4-48.9	2.1-3.7	0-5
F. clavum	QJ1722	6.8-44.2	2.3-4.1	0-4
F. clavum	QJ1722A	6.7-38.0	2.3-3.9	0-4
F. ipomoeae	QJ5111A	14.9-25.7	2.9-3.6	0-5
F. ipomoeae	QJ5211A	8.5-38.3	2.3-4.1	0-5
F. ipomoeae	QJ5111	28.9-48.1	3.6-4.8	0-5
F. ipomoeae	QJ5211	6.3-36.8	2.4-3.9	0-4
F. scirpi	QJ188	5.6-53.3	2.9-4.7	0-6

Table S3. Pathogenicity of *Fusarium* isolates.

Fusarium species complex	*Fusarium* species	Isolated	Host	Pathogenicity
F. incarnatum-equiseti species complex (FIESC)	*F. clavum*	QJ1722	*Themeda triandra* Forssk.	10%-50%
		QJ1722A	*Themeda triandra* Forssk.	33.3%-50%
	F. flagelliforme	QJ18122	*Bromus japonicus* Thunb.	42.8%-100%
		QJ2093	*Capillipedium parviflorum* R.	83%-100%
		QJ16632	*Avena fatua L.*	100%
	F. ipomoeae	QJ5211	*Heteropogon contortus* L.	0%
		QJ5211A	*Heteropogon contortus* L.	10%-22.2%
		QJ5111	*Setaria viridis* L.	50%-100%
		QJ5111A	*Setaria viridis* L.	66.7%-100%
	F. longifundum	QJ513112	*Avena fatua L.*	77.8%-100%
		QJ51112	*Setaria viridis* L.	22.2%-90%
		QJ5112	*Setaria viridis* L.	10%-66.7%
	F. scirpi	QJ188	*Capillipedium parviflorum* R.	33.3-100%
F. oxysporum species complex (FOSC)	*F. oxysporum*	QJ2001622	*Avena fatua* L.	33.3%-100%
F. sambucinum species complex (FSAMSC)	*F. meridionale*	QJ2092	*Capillipedium parviflorum* R.	50%-100%
		QJ2092A	*Capillipedium parviflorum* R.	66.7%-100%
		QJ2081	*Setaria viridis* L.	33.3%-88.9%
	F. sambucinum	QJ203	*Bothriochloa pertusa* L.	0%
		QJ20911	*Avena fatua* L.	10%-55.6%
		QJ20911A	*Avena fatua* L.	10%-60%
F. tricinctum species complex (FTSC)	*F. acuminatum*	QJ1662	*Neotrinia splendens* Trin.	0%
	F. avenaceum	QJ2111	*Brachypodium sylvaticum* Huds.	100%
		QJ418	*Avena fatua* L.	100%
		QJ488A	*Avena fatua* L.	100%
